# Changes in pulse pressure variability during cardiac resynchronization therapy in mechanically ventilated patients

**DOI:** 10.1186/cc5779

**Published:** 2007-04-19

**Authors:** Cornelius Keyl, Jochem Stockinger, Sven Laule, Klaus Staier, Jochen Schiebeling-Römer, Christoph Wiesenack

**Affiliations:** 1 Department of Anesthesiology, Heart Centre Bad Krozingen, Suedring 15, 79189 Bad Krozingen, Germany; 2 Department of Rhythmology, Heart Centre Bad Krozingen, Suedring 15, 79189 Bad Krozingen, Germany; 3 Department of Anesthesiology, University Hospital Regensburg, Franz-Josef-Strauss-Allee 11, 93042 Regensburg, Germany

## Abstract

**Introduction:**

The respiratory variation in pulse pressure (PP) has been established as a dynamic variable of cardiac preload which indicates fluid responsiveness in mechanically ventilated patients. The impact of acute changes in cardiac performance on respiratory fluctuations in PP has not been evaluated until now. We used cardiac resynchronization therapy as a model to assess the acute effects of changes in left ventricular performance on respiratory PP variability without the need of pharmacological intervention.

**Methods:**

In 19 patients undergoing the implantation of a biventricular pacing/defibrillator device under general anesthesia, dynamic blood pressure regulation was assessed during right ventricular and biventricular pacing in the frequency domain (power spectral analysis) and in the time domain (PP variation: difference between the maximal and minimal PP values, normalized by the mean value).

**Results:**

PP increased slightly during biventricular pacing but without statistical significance (right ventricular pacing, 33 ± 10 mm Hg; biventricular pacing, 35 ± 11 mm Hg). Respiratory PP fluctuations increased significantly (logarithmically transformed PP variability -1.27 ± 1.74 ln mm Hg^2 ^versus -0.66 ± 1.48 ln mm Hg^2^; *p *< 0.01); the geometric mean of respiratory PP variability increased 1.8-fold during cardiac resynchronization. PP variation, assessed in the time domain and expressed as a percentage, showed comparable changes, increasing from 5.3% (3.1%; 12.3%) during right ventricular pacing to 6.9% (4.7%; 16.4%) during biventricular pacing (median [25th percentile; 75th percentile]; *p *< 0.01).

**Conclusion:**

Changes in cardiac performance have a significant impact on respiratory hemodynamic fluctuations in ventilated patients. This influence should be taken into consideration when interpreting PP variation.

## Introduction

The respiratory fluctuations of stroke volume and its surrogate, pulse pressure (PP), in mechanically ventilated patients are an expression of the relationship between changes in left ventricular preload and stroke volume. Several studies have found a significant correlation between PP variation or stroke volume variation and the increase in cardiac output caused by fluid loading [1-3]. Therefore, respiratory fluctuations of cardiovascular parameters are accepted measures of cardiac volume responsiveness in mechanically ventilated patients [4].

However, preload is only one determinant of cardiac performance (besides ventricular contractility and afterload properties). Whereas the influence of changes in preload on the variation of stroke volume, PP, or systolic blood pressure (SBP) has been investigated in detail, the extent to which these dynamic measures are modified by changes in ventricular performance is not yet clear. This might be due to the fact that the treatment with inotropic drugs also modifies heart rate and vascular tone, thus making it impossible to study the isolated effect of changes in cardiac performance on dynamic cardiovascular measures.

We used cardiac resynchronization therapy as a model to study the effect of changes in cardiac performance on the variation of hemodynamic variables. Cardiac resynchronization therapy is an accepted therapeutic approach for improving cardiac performance in patients with heart failure associated with an intraventricular conduction disorder [5-7]. In the present study, we compared the acute effects of right ventricular and biventricular pacing on PP fluctuations in mechanically ventilated patients with impaired myocardial function. We were thus able to assess the influence of changes in cardiac contractility on static and dynamic cardiocirculatory parameters without changing heart rate, vascular tone, or intravascular volume status.

## Materials and methods

After obtaining the approval of the local ethics committee and written informed consent, we studied 19 patients (15 men, ages 51 to 78 years) with New York Heart Association class III (18 patients) and class IV (1 patient) heart failure and dyssynchrony between right and left ventricular contractions who were scheduled for the implantation of a combined biventricular pacing/defibrillator device (Contak Renewal; Guidant GmbH, Giessen, Germany).

The patients underwent routine monitoring by means of electrocardiogram (ECG), pulse oximetry, and non-invasive blood pressure monitoring (IntelliVue MP50; Philips Medizin Systeme Böblingen GmbH, Böblingen, Germany). Additionally, the R-R intervals and plethysmographic blood pressure measurement were continuously registered (Task Force Monitor; CNSystems Medizintechnik AG, Graz, Austria).

The patients were prehydrated with 3 ml/kg of an isotonic crystalloid solution, followed by 2 to 3 ml/kg per hour. Anesthesia was induced with 10 to 20 μg of remifantanil and etomidate until loss of consciousness, and tracheal intubation was facilitated by rocuronium 0.6 mg/kg. Anesthesia was maintained by remifantanil 2.5 μg/kg per minute and propofol 0.05 to 0.06 mg/kg per minute as clinically required.

The patients were mechanically ventilated with a constant tidal volume of 7 to 8 ml/kg, a positive end-expiratory pressure of 5 millibars, an inspiratory/expiratory ratio of 1:1, and a respiratory rate of 10 to 12 per minute to maintain an end-tidal pCO_2 _(partial pressure of carbon dioxide) of 35 mm Hg at an FiO_2 _(fraction of inspired oxygen) of 0.5 throughout the entire study period.

A norepinephrine infusion was administered if required to maintain an SBP of 90 mm Hg.

Bipolar electrode catheters were placed in the right atrial appendage, the right ventricle, and via the coronary sinus in a posterior or lateral venous branch.

Three-minute recordings of ECG and arterial blood pressure were performed in a hemodynamic steady state during right ventricular and biventricular pacing. ECG and blood pressure were sampled at 1,000 Hz and stored on the hard disk of a personal computer.

Frequency-domain analysis of SBP and PP variability was performed in accordance with the suggestions of the Task Force of the European Society of Cardiology and the North American Society of Pacing and Electrophysiology [8]. Signals were inspected visually and checked for artifacts and heterotopic beats that would have been removed by interpolation by means of interactive software. Time series were computed with SBP and PP. Stationarity of each period was checked by the reverse arrangement test described by Bendat and Piersol [9]. Data were resampled at 4 Hz using a moving 500-millisecond-wide rectangular window. After substraction of the mean value of the sample data, removal of residual linear trends, and application of a cosine function (Hanning window) to avoid distortions of the estimated spectra [9], discrete Fourier analysis was performed for three 50% overlapping windows, and the results were subsequently averaged. The area under the curve was calculated for the frequency component of respiration (respiratory frequency ± 0.025 Hz).

Additionally, time-domain analysis of PP was performed. The three-minute data files were divided into 7.5-second periods. The difference between the minimum and maximum values of PP, normalized by the mean of the two values and expressed as a percentage, was calculated in each window. The results of the 24 periods were subsequently averaged.

Statistical analysis was performed using commercially available software (SPSS for Windows, version 12.0; SPSS Inc., Chicago, IL, USA). The data were checked for normal distribution by means of the Lilliefors modification of the Kolmogorov-Smirnov test. The results of spectral power analysis were normally distributed after logarithmic transformation. Data are presented as mean ± standard deviation or as median (25th percentile; 75th percentile). Data were compared using the Student *t *test for paired data or the Wilcoxon signed rank test, as appropriate. An α error of 0.05 was considered significant.

## Results

Demographic data and the characteristics of the patients are presented in Table 1. Five patients required norepinephrine up to a dosage of 2 μg/minute intraoperatively to maintain an SBP of 90 mm Hg. The results of the hemodynamic measurements are presented in Table 2. Because the frequency of pacing did not change between right ventricular and biventricular pacing, the mean R-R interval was identical at the two sample points. Systolic, mean, and diastolic blood pressures did not change significantly between right ventricular and biventricular pacing. PP increased by 2 mm Hg during biventricular pacing. This increase, however, did not reach statistical significance (*p *= 0.08).

**Table 1:** Demographic data

Age (years)	69 ± 6
Height (cm)	172 ± 9
Weight (kg)	81 ± 15
Dilated cardiomyopathy (*n*)	8
Ischemic heart disease (*n*)	11
LVEF (percentage)	24 ± 6
Beta-receptor blockers (*n*)	17
ACE inhibitors/Angiotensin II blockers (*n*)	18
Amiodarone (*n*)	7
Diuretics (*n*)	19
Cardiac glycosides (*n*)	7

Data are reported as either mean ± standard deviation or as frequency distributions (*n*). ACE, angiotensin-converting enzyme; LVEF, left ventricular ejection fraction.

**Table 2:** Hemodynamic variables during right ventricular and biventricular pacing

	Right ventricular pacing	Biventricular pacing	*P *value
R-R interval (milliseconds)	864 ± 94	864 ± 93	0.94
Systolic blood pressure (mm Hg)	98 ± 18	100 ± 18	0.35
Mean blood pressure (mm Hg)	75 ± 13	75 ± 14	0.75
Diastolic blood pressure (mm Hg)	65 ± 13	64 ± 13	0.88
Pulse pressure (mm Hg)	33 ± 10	35 ± 11	0.08
Respiratory systolic blood pressure variability (ln mm Hg^2^)	-0.57 ± 1.42	-0.17 ± 1.37	0.002
Respiratory pulse pressure variability (ln mm Hg^2^)	-1.27 ± 1.74	-0.66 ± 1.48	0.002
Pulse pressure variation (percentage)	5.3 (3.1; 12.3)	6.9 (4.7; 16.4)	0.008

Data are reported as mean ± standard deviation or as median (25th percentile; 75th percentile). Results of power spectral analysis are logarithmically transformed.

**Figure 1:**
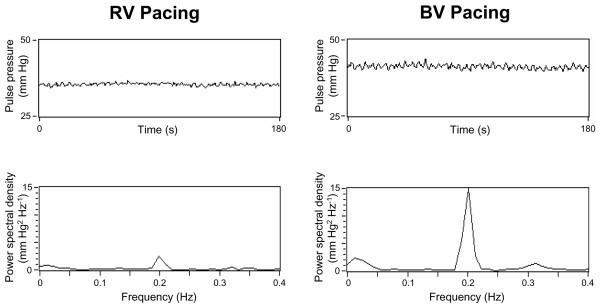
Exemplary three-minute registrations of pulse pressure (PP), recorded during right ventricular and biventricular pacing, and their related power spectra. The patient was ventilated at a frequency of 0.2 Hz. In this patient, mean PP and the respiratory fluctuations of PP increased markedly during biventricular pacing. BV, biventricular; RV, right ventricular.

Exemplary registrations of PP, recorded during right ventricular and biventricular pacing, and their related power spectra are demonstrated in Figure 1. The results of the frequency-domain and time-domain analyses are reported in Table 2. The area under the curve in the respiratory frequency component of SBP and PP increased significantly (*p *< 0.01) during biventricular pacing, with a 1.5-fold increase in the geometric mean of SBP variability (right ventricular pacing, 0.563 mm Hg^2^; biventricular pacing, 0.844 mm Hg^2^) and a 1.8-fold increase in the geometric mean of PP variability (right ventricular pacing, 0.281 mm Hg^2^; biventricular pacing, 0.516 mm Hg^2^). The analysis of PP variation in the time domain, determined as the difference between the highest and lowest values and normalized by the mean value, revealed a 1.3-fold increase from 5.3% to 6.9% during biventricular pacing (*p *< 0.01).

## Discussion

Respiratory fluctuations in PP during mechanical ventilation are an expression of respiratory changes in left ventricular stroke volume: due to a decrease in right ventricular preload and an increase in right ventricular afterload, right ventricular stroke volume decreases during inspiration. Left ventricular stroke volume decreases with a delay of one to two heartbeats and is additionally modified by a variety of factors, such as a decrease in left ventricular afterload during inspiration [10-12].

Most studies dealing with PP variation have focused on the influence of the volume status on respiratory fluctuations: an increase in preload is related to a rightward shift of the cardiac operating point on the Frank-Starling curve with the consequence that a patient who is operating on the steep portion of the curve may operate on the flat portion. This change in the position on the Frank-Starling curve is related to a decrease in the respiratory fluctuation of PP.

In contrast to previous studies, we did not focus on the influence of the intravascular volume on the position of the cardiac operating point on the Frank-Starling curve, but on the influence of cardiac performance on the slope of the preload/stroke volume relationship. A decrease in ventricular contractility decreases the slope of the relationship between end-diastolic volume and stroke volume [13]. Thus, the respiratory fluctuations of stroke volume and PP should decrease in the failing heart in mechanically ventilated patients. Conversely, an improvement in cardiac performance should create an increase in the respiratory fluctuations of PP: a patient with heart failure who operates on a flattened Frank-Starling curve may operate on a much steeper portion of the new curve.

Our results confirm this physiological model. The respiratory fluctuations of PP increased significantly during biventricular pacing in our patients with severely impaired myocardial function.

### The influence of biventricular pacing on the dynamic behavior of PP

In previous studies, PP variation derived by time-domain analysis was markedly decreased when compared to patients with normal or moderately impaired myocardial function [3,14]. Reuter and colleagues [1] compared stroke volume variation of patients with normal left ventricular function with that of patients with impaired left ventricular function and found that stroke volume variation was decreased in patients with an ejection fraction of less than 35%, but without statistical significance. Both patient groups with normal myocardial function and with impaired myocardial function showed significant fluid responsiveness (that is, a decrease in PP variation after fluid loading) [1]. In our patients with severely impaired cardiac function, PP variation remained (even during biventricular pacing) below the threshold value indicating fluid responsiveness, which was determined to be 10% [14]. Because our patients were suffering from advanced drug-refractory heart failure, we did not test the effect of fluid loading on PP variability.

In addition to time-domain analysis, we assessed blood pressure variability by spectral analysis and were thus able to analyze the influence of ventilation on blood pressure at the specific frequency of respiration. Respiratory fluctuations in SBP are less closely related to changes in stroke volume than are respiratory fluctuations in PP [10,12]. Nevertheless, the respiratory fluctuations of SBP behaved comparably to those of PP in our patients.

Previously, in adults anesthetized with propofol, we observed that the respiratory fluctuations of blood pressure were superimposed by major fluctuations, which were located at a significantly lower frequency component than the mechanical ventilation or the Mayer waves [15]. In these patients, the difference between maximum and minimum SBPs (taken as a measure of volume responsiveness) could have provided a misleading result not related to the respiratory changes in arterial pressure if the time interval had been much longer than the respiratory cycle length. Our results do not confirm this concern: we determined SBP and PP variation at a time interval of 7.5 seconds (in accordance with previous studies [3]) and observed a similar behavior of fluctuations of SBP and PP calculated in the time domain and in the frequency domain.

The interaction of respiratory changes in stroke volume and intrathoracic and transpulmonary pressures is of major importance when interpreting respiratory fluctuations of blood pressure [16]. A previous study indicated that an increase in tidal volume was associated with an increase in PP variation [14]. Other investigators found that the stroke volume variation, assessed during ventilation with a large tidal volume (15 ml/kg), was a good predictor of volume responsiveness [17], whereas our group had inconsistent results when ventilating patients with smaller tidal volumes [3,18]. In the present study, we ventilated patients with a tidal volume of 7 to 8 ml/kg body weight at a respiration rate of 10 to 12 per minute. Larger tidal volumes would have required a decrease in respiration rate in order to maintain normoventilation. It is well known that in the awake, spontaneously breathing human the respiratory hemodynamic fluctuations increase at lower breathing frequencies (despite constant tidal volume) and have a maximum of approximately 0.1 Hz (that is, the frequency of the spontaneously occurring Mayer waves) [19-21]. Until now, whether this phenomenon also occurs under general anesthesia has not been investigated. In a previous study, we found that the hemodynamic fluctuations of approximately 0.1 Hz were markedly depressed under propofol anesthesia [15]. However, it remains to be examined whether not only the tidal volume, but also the respiratory frequency, is relevant when analyzing respiratory hemodynamic fluctuations during anesthesia in mechanically ventilated patients.

### The influence of biventricular pacing on static changes in PP

Several studies reported an improvement in dP/dt_max _as a measure of isovolumic systolic function and in PP as a surrogate of stroke volume during biventricular pacing in patients with heart failure and intraventricular conduction delay [5,22]. Consequently, these parameters are frequently used to assess the optimal position of the left ventricular electrode and the stimulation configuration [23]. The PP of our patients during right ventricular pacing is comparable to that reported by other authors during baseline conditions [5]. However, we found a modest increase in mean PP during biventricular pacing of approximately 2 mm Hg, which was not significant. Other authors have reported an increase of 4 to 8 mm Hg [6,22]. It is not clear whether these differences are related to the electrode positioning, regional differences in ischemic lesions, or methodological differences in blood pressure recording. Investigators who determined cardiac output invasively or by echocardiography found a median increase of 8% and mean increases of 10% and 15%, respectively, during biventricular pacing [7,24,25]. Because our patients met the characteristics of patients who typically show an improvement in cardiac performance during biventricular pacing, we avoided the potential risk that is related to the invasive measurements of cardiac output.

### Limitations of the study

The absence of invasive measurement of cardiac output for the above-mentioned reason may be a limitation of the study. We measured beat-to-beat blood pressure by a finger plethysmographic method, which is calibrated by the oscillometric measurement of blood pressure (Task Force Monitor; CNSystems). This methodology showed satisfactory precision in the assessment of blood pressure changes compared to intra-arterial measurement [26,27]. Pinna and colleagues [28] observed sufficient agreement in the frequency band of respiration in patients with chronic heart failure when comparing the power spectra of blood pressure recorded invasively and by the finger plethysmographic method, respectively. Nevertheless, we cannot rule out that results assessed by finger plethysmographic measurements are not fully comparable to those of studies using invasive blood pressure measurement.

## Conclusion

Our findings indicate that changes in cardiac performance have a significant influence on respiratory fluctuations in PP. This interaction should be considered when interpreting PP variation in the clinical setting. Our results suggest, furthermore, that respiratory PP variability is a parameter that is much more sensitive to an improvement in systolic cardiac performance than changes in the mean value of PP in mechanically ventilated patients with heart failure associated with an intraventricular conduction disorder. Whether this parameter might be helpful in guiding the positioning of electrodes and optimizing the configuration of biventricular stimulation in mechanically ventilated patients remains to be determined.

## Key messages

• Cardiac performance has a considerable impact on the respiratory fluctuations of pulse pressure in mechanically ventilated patients with heart failure.

• Cardiac resynchronization therapy is associated with minor changes in blood pressure but with major changes in respiratory fluctuations of pulse pressure in mechanically ventilated patients.

## Abbreviations

ECG = electrocardiogram; PP = pulse pressure; SBP = systolic blood pressure.

## Competing interests

The authors declare that they have no competing interests.

## Authors' contributions

CK designed the study, processed and analyzed the data, and wrote the manuscript. JS contributed to the study design and interpretation of data. SL collected the clinical data and revised the manuscript critically. KS contributed to the acquisition of data. JS-R collected the clinical data and contributed to data interpretation. CW was involved in data interpretation and drafting the manuscript. All authors read and approved the final manuscript.
